# Neutron activation data for the analysis of archaeological and geological hematite in Missouri and Illinois, U.S.A.

**DOI:** 10.1016/j.dib.2023.109787

**Published:** 2023-11-07

**Authors:** Daniel E. Pierce, Rachel S. Popelka-Filcoff

**Affiliations:** a Missouri State University (Center for Archaeological Research, Missouri State University, 901 South National Avenue, Springfield, MO 65897, USA; b University of Melbourne (School of Geography, Earth, and Atmospheric Sciences, University of Melbourne, Victoria 3010, Australia

**Keywords:** Ochre, Geochemistry, Provenance, Prehistory, North America

## Abstract

Hematite, and more broadly ochre, have long been used by humans throughout history for a variety of applications. In prehistoric North America the use of hematite is as old as its first migrants. This data article includes data related to the analysis of archaeological hematite in the American Bottom region in Missouri and Illinois, U.S.A. The data include archaeological samples dating from the Late Archaic Period (3000 – 1000 BCE) to the Middle Woodland Period (150 BCE – 400 CE) from nine sites within the general St. Louis area (*n* = 69), as well as 29 samples from Verkamp Rockshelter in the iron-rich region of the Meramec River Valley. The data is supplemented with geological samples collected from five raw deposits in southeastern Missouri (*n* = 70). Data was acquired through Neutron Activation Analysis to assess provenance of all archaeological samples. Following the irradiation of samples, data was normalized for iron content before statistical analysis. A variety of multivariate statistical routines, including principal component and cluster analyses were then employed to assess possible origin locales for all archaeological samples. This data article also includes maps, tables, and figures to assist in understanding the analysis conducted.

Specifications Table

**Table:** 

Subject	Social Sciences: Archaeology
Specific subject area	Archaeometry
Data format	Raw and Analyzed
Type of data	Data, maps, image, tables, and figure
Data collection	Instrumental Neutron Activation Analysis; Elemental data was collected on high-resolution germanium detector and analyzed with Microsoft Excel and GAUSS 8.0 statistical analysis software
Data source location	Samples were collected from Various archaeological sites in Missouri and Illinois. Data was produced at the Archaeometry Laboratory of the University of Missouri Research Reactor
Data accessibility	Repository name: Mendeley Data Data identification number: DOI:10.17632/977X99yvc4.2 Direct URL to data: https://data.mendeley.com/datasets/977x99yvc4/2
Related research article	Pierce, Daniel E., and Rachel S Popelka-Filcoff (2023) Regional ochre procurement in the prehistoric American Bottom. *Journal of Archaeological Science: Reports* (51): 104191. DOI:10.1016/j.jasrep.2023.104191.

## 1 Value of the Data

• These data are the first NAA data produced for archaeological ochres in Missouri. It is significant in that few studies have addressed provenance of ochre artifacts beyond their use as pigments, and as such may be of particular interest to those interested in the assessment of provenance for ochre artifacts in the region and elsewhere in North America.
• For re-use of these data, researchers may compare newly collected compositional (NAA) data from other artifacts to the data presented here to assess potential origin locales. It is especially useful in that it contains reference sample data from multiple geological sources, as well as an array of samples from many sites, including one major site in an iron rich region of Missouri.
• Data can also be compared to data from other regions to better understand the expected range of compositional variation that may exist within sources and regions.

## 2 Data Description

**Fig. 1:**
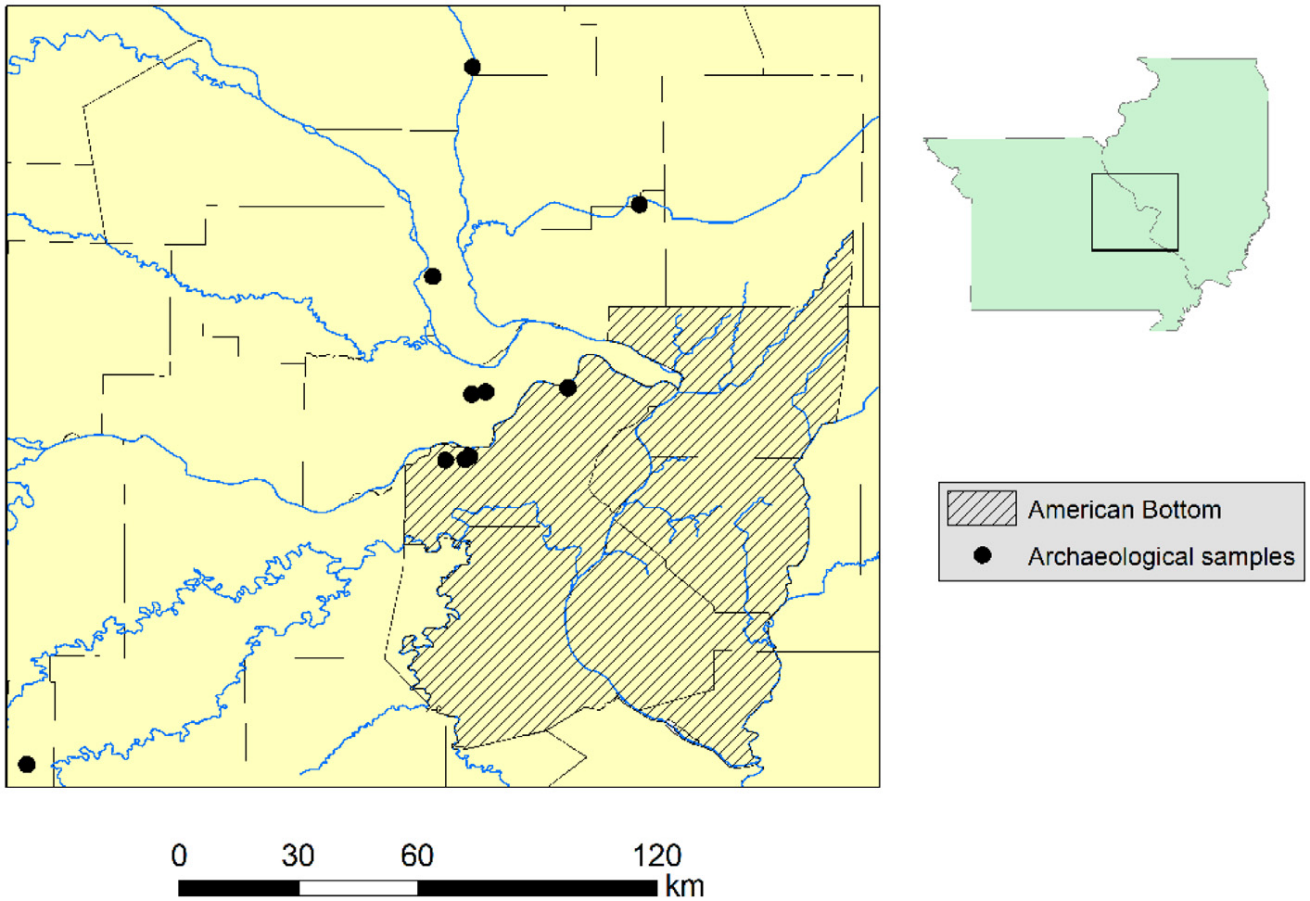
Geographical provenience of archaeological samples.

**Fig. 2:**
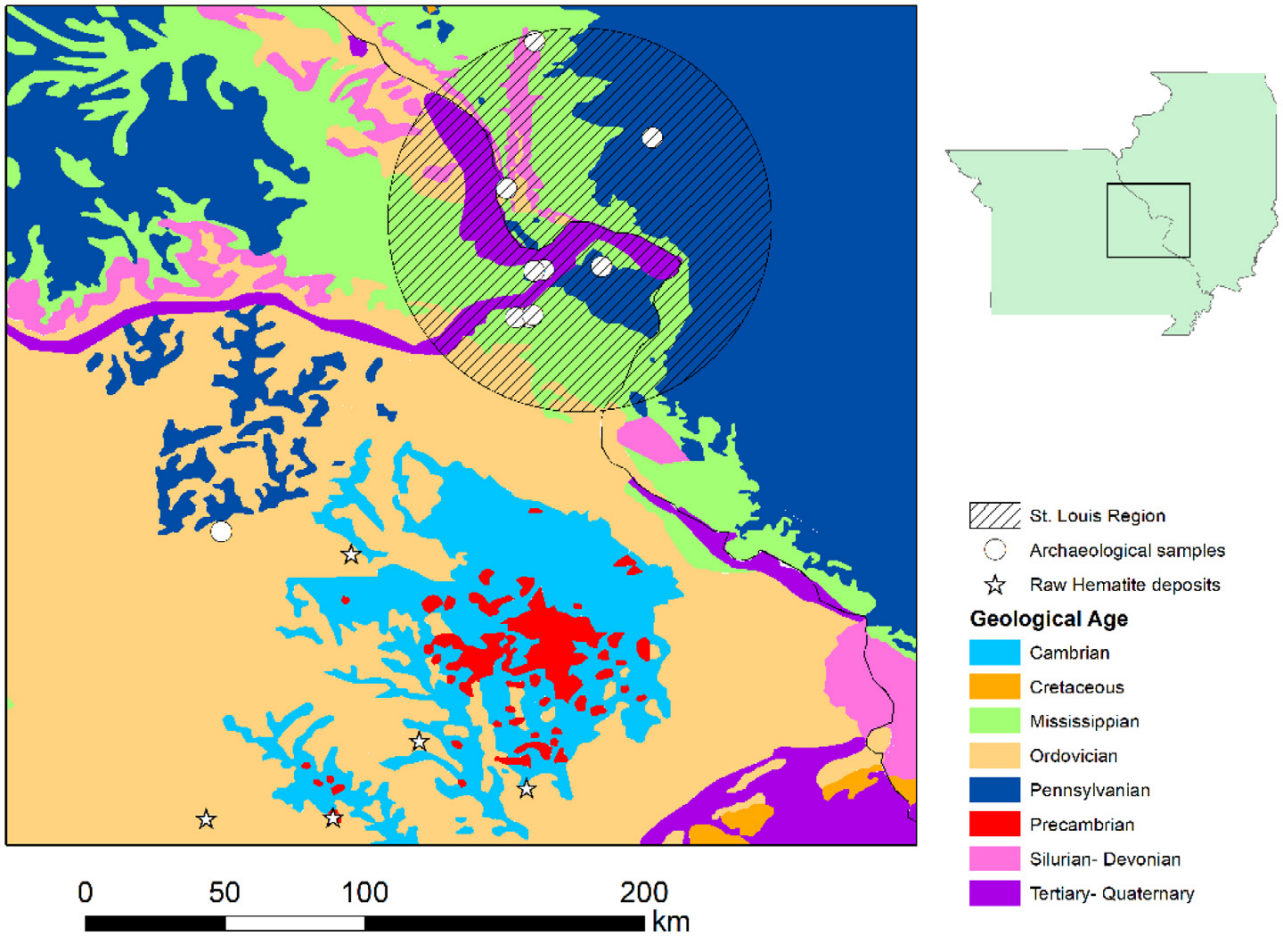
Geology of region.

**Fig. 3:**
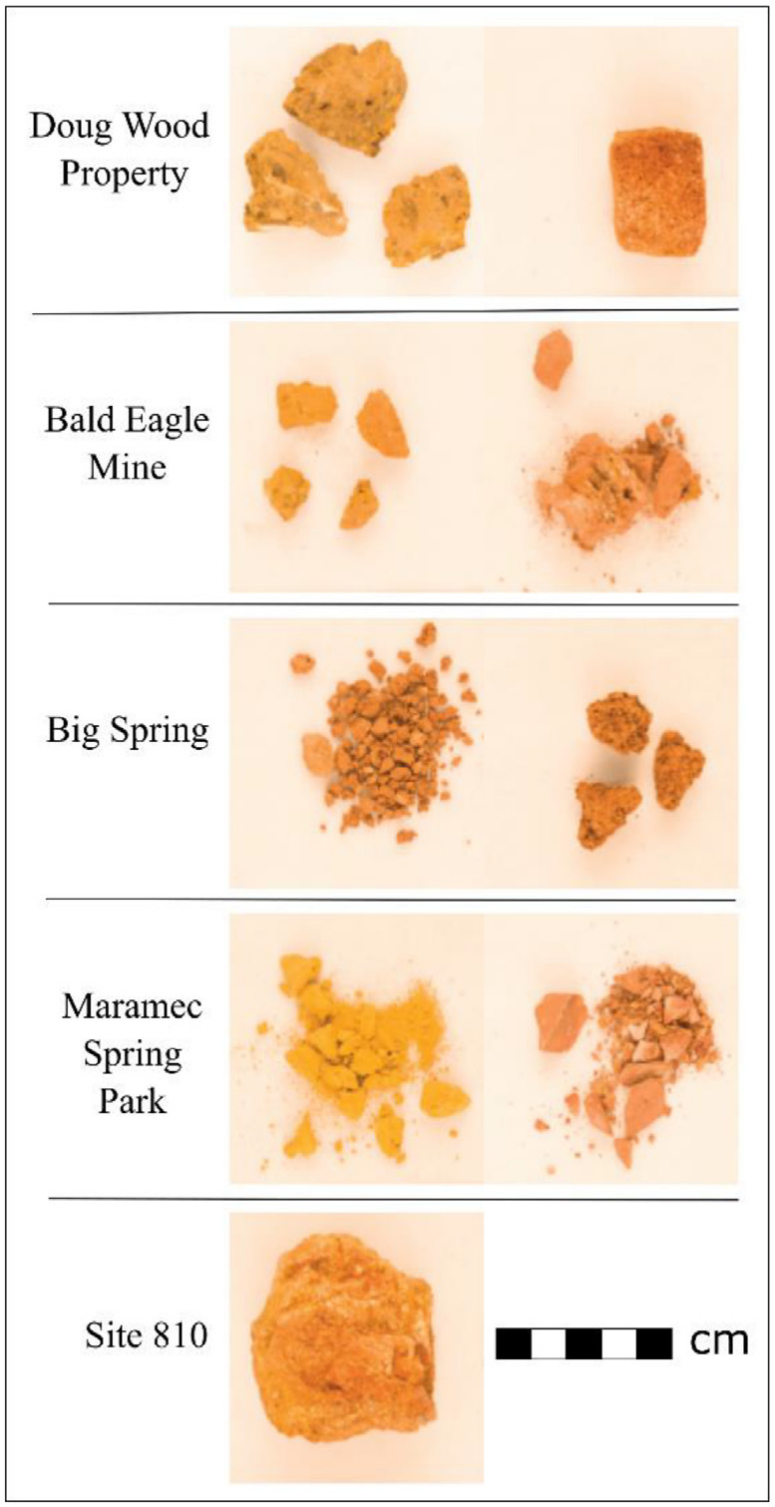
Unprocessed geological material samples from five sites in southeastern Missouri (photos by Venera May; Samples from Popelka-Filcoff et al. 2006 and 2007).

**Fig. 4:**
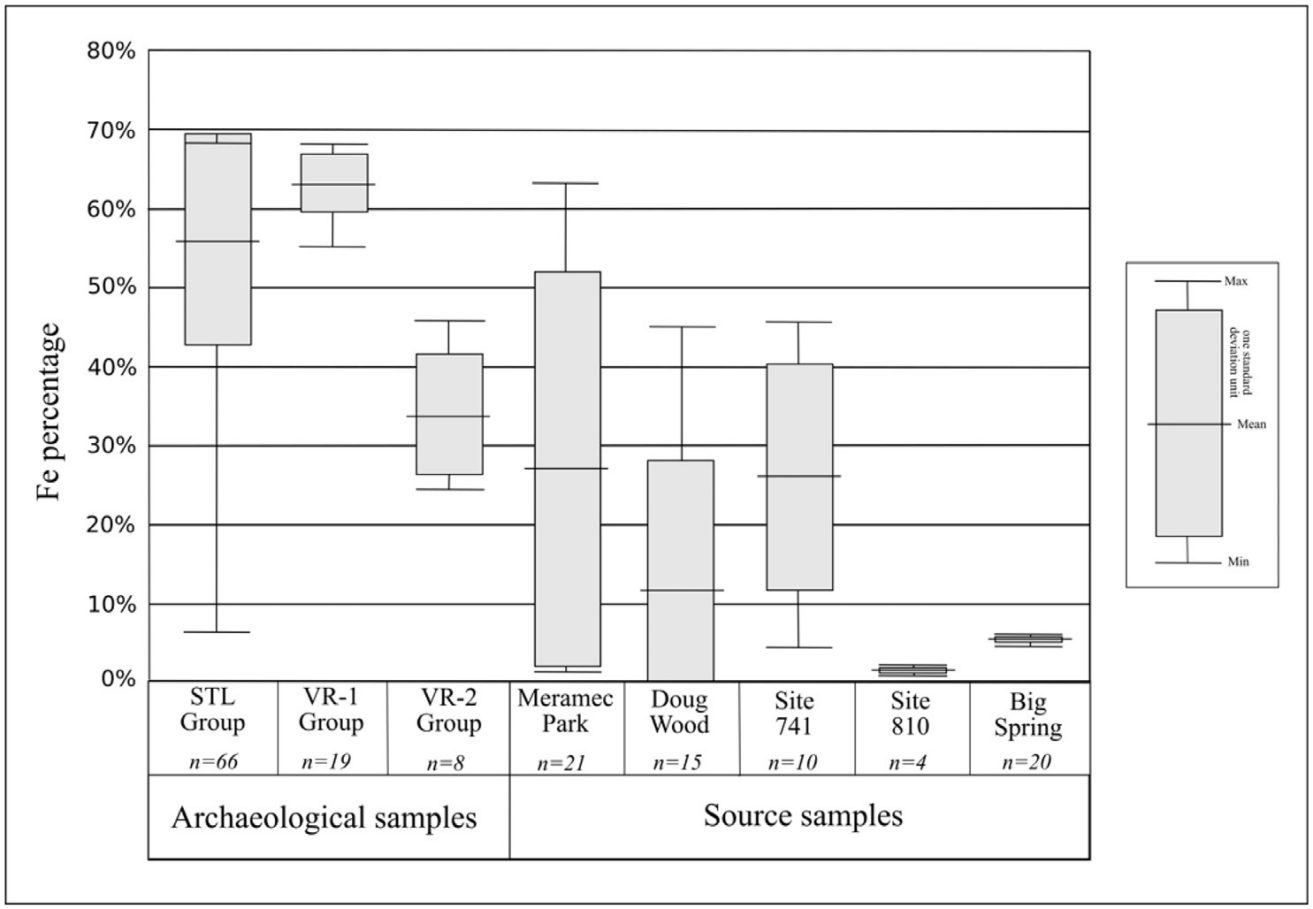
Iron content variation in the different compositional groups.

A map of the American Bottom region shows the locations of sites included in this study (Fig. 1, Table 1). A map of the local geology is also included (Fig. 2), as well as photographs of raw material collection (Fig. 3). Ochre samples were analyzed by neutron activation analysis at the University of Missouri Research Reactor (MURR) and are explored here [1: Supplement 1- raw data]. The results of these analysis are explored through various mathematical analyses, including Fe-normalization (Fig. 4) [1: Supplement 2- Fe normalized data], chondrite normalization [1: Supplement 3-chrondite normalized data], and several multivariate statistical routines to both characterize and discriminate the ochre samples (Tables 2 and 3). Ultimately, data and analyses were used to address differences between archaeological samples and raw materials, assisting in questions of potential provenance. All data is curated and publicly available in the online Mendeley Data repository [1], and is associated with the previously published work by the authors [2,3].

**Table 1:** Origin of samples included in study.

	Site	# of samples
Archaeological samples	Gateway	2
	Hayden	2
	Lawless	2
	Priest	7
	Solto	7
	Truman Road	31
	Snyders	6
	Loy	6
	Wheeler	6
	Verkamp Rockshelter	29
Reference samples	Maramec Springs Park	21
	Doug Wood	15
	Bald Eagle Mine	10
	Site 810	4
	Big Spring	20

**Table 2:** Principal Component values representing 90 % of the total variation collectively.

Variable	Average	PC1	PC2	PC3	PC4	PC5
As	162.374	0.219	0.462	-0.154	0.503	-0.308
La	54.7991	0.264	-0.062	0.020	-0.289	-0.270
Nd	74.7031	0.276	-0.096	0.117	-0.135	-0.164
Sm	21.5784	0.293	-0.125	0.159	0.028	-0.067
U	47.7619	0.243	0.200	0.155	0.158	0.002
Yb	5.95835	0.274	-0.193	0.224	0.197	0.166
Ce	120.268	0.274	-0.083	0.028	-0.235	-0.170
Co	125.877	0.225	-0.057	-0.560	-0.035	0.319
Cr	156.956	0.112	0.389	-0.167	-0.223	-0.020
Eu	3.96479	0.298	-0.199	0.215	0.095	-0.014
Sb	16.0027	0.248	0.400	0.155	-0.078	-0.001
Sc	19.9315	0.189	-0.028	0.014	-0.220	0.394
Th	7.90774	0.123	0.179	-0.007	-0.514	0.195
Zn	309.778	0.201	-0.171	-0.363	-0.183	-0.537
Al	104958	0.116	0.205	0.117	-0.039	0.260
Dy	13.7326	0.286	-0.218	0.214	0.150	0.118
Mn	3878.7	0.253	-0.271	-0.462	0.275	0.208
Na	1233.59	0.146	0.081	0.154	0.078	0.016
V	767.154	0.162	0.296	-0.148	0.083	0.184
Eigenvalues:		4.166	1.287	0.476	0.230	0.192
% variation explained:		59.04%	18.24%	6.75%	3.25%	2.72%

**Table 3:** Average elemental values for each compositional group.

	STL-1 (*n* = 66)			VR-1 (*n* = 19)			VR-2 (*n* = 8)		
	Mean	St. Dev	CV	Mean	St. Dev	CV	Mean	St. Dev	CV
As	370.0	348.4	0.94	1444	2706	1.87	130.7	350.4	2.68
La	259.5	707.1	2.72	67.64	51.95	0.77	10.09	4.626	0.46
Nd	301.0	599.9	1.99	55.47	54.60	0.98	12.93	8.653	0.67
Sm	83.66	136.9	1.64	15.39	9.337	0.61	3.503	2.486	0.71
U	145.4	139.6	0.96	87.79	60.46	0.69	7.864	2.634	0.33
Yb	25.82	48.70	1.89	3.123	3.132	1.00	1.033	0.718	0.70
Ce	530.4	1280	2.41	140.0	106.5	0.76	21.78	11.39	0.52
Co	288.7	302.5	1.05	465.7	635.8	1.37	40.61	88.42	2.18
Cr	162.4	245.8	1.51	852.5	877.0	1.03	79.61	76.86	0.97
Eu	17.89	28.73	1.61	2.263	1.955	0.86	0.733	0.707	0.97
Sb	44.81	45.07	1.01	62.03	45.85	0.74	3.534	3.191	0.90
Sc	47.28	41.33	0.87	24.48	16.80	0.69	5.856	3.140	0.54
Th	8.897	5.591	0.63	19.60	13.68	0.70	5.748	3.947	0.69
Zn	1068	1190	1.12	283.0	305.2	1.08	108.4	275.0	2.54
Al	146060	89261	0.61	180535	62984	0.35	52080	29049	0.56
Dy	60.22	98.49	1.64	8.623	8.034	0.93	2.326	2.145	0.92
Mn	20413	32489	1.59	3437	2822	0.82	665.0	1246	1.87
Na	2193	1805	0.82	1368	942.9	0.69	369.9	119.7	0.32
V	1190	1347	1.13	2617	1900	0.73	290.0	237.5	0.82

## 3 Experimental Design, Materials And Methods

### 3.1 Experimental Design, Materials, And Methods

#### 3.1.1 Study Area

The samples included in this study consist of 98 archaeological samples and 70 geological samples (Table 1). Of these, 38 of the archaeological ochre samples were originally included as part of a previous study restricted to the St. Louis region, and focused on the Truman Road site, in St. Charles County, Missouri [3]. The additional archaeological hematite samples included in this study are a mixture of samples (*n* = 31) collected from five more sites on either side of the Mississippi River within the St. Louis Region, and 29 samples collected from Verkamp Rockshelter in Phelps County, Missouri. The most significant of any site in this study, Verkamp Rockshelter is a multicomponent site dating from the Archaic to the Woodland Period [4]. Given the site's size, continuity, and comparatively thorough study, it has been critical to establishing the regional cultural sequence [5]. On the other hand, raw material deposit samples included here were originally analyzed independently of archaeological samples by Popelka-Filcoff [6].

#### 3.1.2 Sample Description/Selection

The samples in this study were selected to understand regional ochre procurement patterns. Previously, studies had suggested that St. Louis area archaeological ochres had originated in the Meramec River Valley. For this reason. Archaeological samples from this area (Verkamp Rockshelter) were also included in this study. St. Louis region samples were selected opportunistically based upon what was available, resulting in a range of sample sizes from two to 30 per site. Verkamp Rockshelter, on the other hand, was selected to represent the Fe-rich Meramec River Valley. Within this site, only a portion of each ochre sample was analyzed to ensure the preservation of the overall assemblage and to be able to consider specimens from multiple vertical and horizontal contexts to examine the full scope of site-wide compositional variability. Geological samples were previously selected based upon archaeological surveys of historical mines. All samples were collected from the rims and sides of mine pits to avoid potential historical disturbances within pits [7]. In total, five deposits were sampled through southeastern Missouri (Figs. 2 and 3). This area is known for its iron-rich deposits and was utilized in both prehistoric and historic times for iron extraction [7], [8], [9], [10], [11].

#### 3.1.3 Sample Preparation for NAA

To prepare for neutron activation analysis, all samples were first ground into a powder using an agate mortar and pestle for sample homogenization. Samples were then weighed and placed in vials for irradiation and subsequent decay for variable lengths of time. For short counts, 60 mg of powdered sample was placed in a 1.2 ml high-density polyethylene tube for 5s of irradiation at a thermal flux of approximately 8.0 × 10^13^ neutrons cm^−2^ s^−1^. These samples were then allowed to decay for 25 min, and counted for 720 s to determine concentrations for Al, Ba, Ca, DY, K, Mn, Na, Ti, and V. For mid-count and long-count measurements, a second 60 mg of sample powder was placed in a high purity quartz vial and irradiated for 24 h at a thermal neutron flux of approximately 5.2 × 10^13^ neutrons cm^−2^ s^−1^. After 7 days of decay, the “mid” count was acquired for 2000 s to determine As, Lu, Nd, Sm, U, and Yb. Finally, after 3 weeks, the “long” count was acquired for 10,000 s to determine Ce, Co, Cr, Cs, Eu, Fe, Hf, Ni, Rb, Sb, Sc, Sr, Ta, Tb, Th, Zn, and Zr. Standards used for NAA were NIST SRM 1633a (fly ash) and SRM 688 (basalt), with quality control standards of NIST SRM 278 (Obsidian Rock) and Ohio red clay [12]. The resulting complete NAA data are available in Appendix A [1].

#### 3.1.4 Fe normalization

With the analysis of hematite, an additional correction is necessary to account for the trace elemental relationship with iron and modeling the contribution of Fe-oxides and -hydroxides as well as to determine the elements correlated with Fe in ochre [7,13]. This analysis can inform analysts as to which are the most useful elements for discriminating sources and can be approached in multiple ways [14]. One method may be most effective with one assemblage but be less so with another [1]. For the current study, using a Pearson's correlation only, elements which are positively correlated with Fe (*p* > 0) were used in further statistical analysis. After eliminating the negatively correlated elements, all data were then normalized to compensate for the widely ranging Fe concentrations. This was accomplished by the Fe- correction factors used in earlier studies, including the modelling the Fe-oxide concentration and accounting for the overall Fe concentration [7,^13^,15]. The resulting corrected values for each element were used for statistical analyses [1: Appendix B].

#### 3.1.5 Chondrite Normalization

Chondrite normalization was calculated based on a simple ratio of the element to the chondrite value as published by McDonough and Sun [16]. Elemental NAA values were not complete for all elements to compare; therefore, observation of overall trends could not be analyzed, particularly around any potential Eu anomalies. However, these data provide overall trends to evaluate with and across sites and sample groups. Chondrite normalized values are provided in [1: Appendix C].

#### 3.1.6 Multivariate Statistical Analyses

Using MURR's in-house GAUSS software, multiple multivariate statistical analyses were conducted. These include a base-10 logarithm to characterize the sample as well as determine compositional differences and similarities reflective of differential provenance, including comparing archaeological samples to reference materials collected directly from deposits previously. Once the data were corrected to account for Fe influence, principal component analyses were conducted to explore the overall composition of the assemblage collectively (Table 2). Cluster analyses were also considered. Discussion of these common geochemical analyses can be found elsewhere [17], [18], [19], [20]. Following general characterization, samples were explored through the visual inspection of bivariate plots. This inspection was performed to identify compositional groups irrespective of site of collection, but also to group samples by region and site to determine potential relationships between provenance and provenience. Following the discrimination of compositional groups, statistics were calculated to explore overall group homogeneity (Table 3) and Fe content (Fig. 4). Finally, archaeological samples were compared to raw materials, the differentiation of which had previously been established [4].

## Limitations

Not applicable

## Ethics Statement

This work abides by all ethical standards and has not included the involvement of human subjects, animal experiments, nor any data collected from social media platforms.

## CRediT authorship contribution statement

**Daniel E. Pierce:** Conceptualization, Methodology, Data curation, Writing – original draft, Writing – review & editing. **Rachel S. Popelka-Filcoff:** Methodology, Writing – original draft, Writing – review & editing.

## Data Availability

NAA Hematite Data (Missouri and Illinois) (Original data) (Mendeley Data).

## Appendix Supplementary materials

Image, application 1

Image, application 2

Image, application 3
