# Supplementary material for: Neutron activation data for the analysis of archaeological and geological hematite in Missouri and Illinois, U.S.A
Source: Data Brief. 2023 Nov 7;51:109787. doi: 10.1016/j.dib.2023.109787 (PMC10694049; doi:10.1016/j.dib.2023.109787)
Supplement: Supplementary file 3 [file mmc3.docx]

SI Table 3: Chondrite normalised NAA data for samples analysed in this study; denoted by geological or archaeological type. Data normalised to McDonough and Sun 1995.

| **Location** | **Site ID** | **Type** | **La/Chon** | **Ce/Chon** | **Nd/Chon** | **Sm/Chon** | **Eu/Chon** | **Tb/Chon** | **Dy/Chon** | **Yb/Chon** | **Lu/Chon** |
| --- | --- | --- | --- | --- | --- | --- | --- | --- | --- | --- | --- |
| Meramac Park | A-I-1 | Geological | 2.18 | 2.15 | 6.66 | 1.73 | 0.53 |  |  |  |  |
| Meramac Park | A-I-2 | Geological | 1.89 | 1.55 |  | 0.98 | 0.42 |  |  |  | 0.99 |
| Meramac Park | A-I-3 | Geological | 6.11 | 5.57 | 4.77 | 2.58 | 0.95 |  |  | 1.11 | 0.89 |
| Meramac Park | A-I-4 | Geological | 10.76 | 9.17 | 12.54 | 6.30 | 2.31 | 2.52 | 1.09 | 1.38 | 0.98 |
| Meramac Park | A-II-1 | Geological | 4.88 | 4.49 |  | 4.44 | 0.89 |  | 1.57 | 2.71 | 5.00 |
| Meramac Park | A-II-2 | Geological | 8.14 | 8.45 | 8.84 | 5.92 | 1.37 |  | 2.37 | 2.47 | 5.48 |
| Meramac Park | A-II-3 | Geological | 9.50 | 9.83 | 9.22 | 7.89 | 2.03 |  | 5.30 | 6.80 | 6.61 |
| Meramac Park | A-II-4 | Geological | 1.31 | 1.53 |  | 1.27 |  |  |  |  | 2.96 |
| Meramac Park | A-II-5 | Geological | 5.65 | 5.51 |  | 4.50 | 1.22 |  | 0.29 | 1.31 | 1.26 |
| Meramac Park | A-III-1 | Geological | 2.34 | 2.59 |  | 4.01 | 1.73 |  | 2.15 | 3.00 | 2.01 |
| Meramac Park | A-III-2 | Geological | 18.55 | 19.24 | 28.69 | 14.00 | 4.83 |  | 4.47 | 1.77 | 1.39 |
| Meramac Park | A-III-4 | Geological | 41.68 | 39.61 | 40.06 | 28.13 | 14.64 | 14.29 | 13.01 | 9.64 | 9.54 |
| Meramac Park | A-IV-1 | Geological | 45.75 | 31.10 | 24.63 | 19.45 | 7.59 |  | 7.72 | 4.83 | 7.61 |
| Meramac Park | A-IV-2 | Geological | 44.98 | 28.94 | 32.71 | 19.57 | 9.15 |  | 7.72 | 5.20 | 4.51 |
| Meramac Park | A-IV-3 | Geological | 33.85 | 28.79 | 12.22 | 8.80 | 3.17 |  | 2.03 |  |  |
| Meramac Park | A-IV-4 | Geological | 34.56 | 29.42 | 36.70 | 28.37 | 12.40 | 5.81 | 10.98 | 5.75 | 12.55 |
| Meramac Park | A-IV-5 | Geological | 5.87 | 4.54 |  | 3.30 | 1.13 |  | 1.22 | 1.36 | 1.26 |
| Meramac Park | A-V-1 | Geological | 3.50 | 3.22 |  | 2.50 | 0.67 | 1.04 | 0.41 |  |  |
| Meramac Park | A-V-2 | Geological | 50.31 | 44.52 | 25.34 | 17.22 | 6.74 |  | 3.66 | 1.33 | 0.00 |
| Meramac Park | A-V-3 | Geological | 10.46 | 11.52 |  | 11.84 | 1.66 | 0.00 | 1.63 |  |  |
| Meramac Park | A-V-5 | Geological | 21.08 | 20.08 | 31.12 | 8.30 | 2.86 |  | 1.63 |  |  |
| Doug Wood Property | B-I-1 | Geological | 139.62 | 123.86 | 61.55 | 39.15 | 18.98 | 20.72 | 19.92 | 20.82 | 19.48 |
| Doug Wood Property | B-I-2 | Geological | 147.90 | 131.92 | 117.73 | 42.19 | 20.33 | 18.77 | 18.29 | 23.41 | 20.33 |
| Doug Wood Property | B-I-3 | Geological | 55.99 | 19.16 | 10.42 | 4.36 | 1.79 | 1.81 | 1.63 | 1.96 | 3.22 |
| Doug Wood Property | B-I-4 | Geological | 147.47 | 133.82 | 74.86 | 42.36 | 20.34 | 22.46 | 19.92 | 19.46 | 20.05 |
| Doug Wood Property | B-I-5 | Geological | 145.06 | 132.21 | 70.08 | 41.55 | 20.49 | 21.13 | 20.33 | 21.36 | 19.82 |
| Doug Wood Property | B-II-1 | Geological | 32.94 | 27.48 | 33.78 | 29.02 | 16.69 | 15.35 | 15.45 | 12.92 | 6.15 |
| Doug Wood Property | B-II-2 | Geological | 67.38 | 41.73 | 23.35 | 24.15 | 12.43 | 16.04 | 17.89 | 17.13 | 21.28 |
| Doug Wood Property | B-II-3 | Geological | 58.16 | 35.44 | 24.67 | 21.99 | 11.54 | 15.21 | 9.76 | 9.64 | 11.85 |
| Doug Wood Property | B-II-4 | Geological | 31.16 | 20.39 | 24.32 | 19.73 | 8.21 | 9.00 | 6.10 | 5.80 | 9.14 |
| Doug Wood Property | B-II-5 | Geological | 86.57 | 35.45 | 74.20 | 54.67 | 26.71 | 31.58 | 20.73 | 20.12 | 29.15 |
| Doug Wood Property | B-III-1 | Geological | 41.97 | 17.76 | 11.33 | 4.80 | 1.96 | 1.94 | 1.63 | 2.01 | 2.69 |
| Doug Wood Property | B-III-2 | Geological | 55.32 | 275.74 | 14.95 | 6.84 | 2.96 | 2.27 | 1.63 | 2.31 | 1.67 |
| Doug Wood Property | B-III-3 | Geological | 42.07 | 16.73 | 13.71 | 5.17 | 2.06 | 2.05 | 2.03 | 2.10 | 2.22 |
| Doug Wood Property | B-III-4 | Geological | 49.32 | 17.98 | 11.65 | 4.90 | 1.87 | 2.58 | 2.03 | 1.86 | 3.54 |
| Doug Wood Property | B-III-5 | Geological | 92.25 | 25.62 | 22.30 | 7.12 | 2.98 | 1.61 | 1.63 | 2.10 | 1.70 |
| Site 741 | C-I-1 | Geological | 60.89 | 40.00 | 27.12 | 14.09 | 6.30 |  | 4.47 | 4.96 | 4.57 |
| Site 741 | C-I-4 | Geological | 46.01 | 26.93 | 18.50 | 10.49 | 4.28 | 7.56 | 3.66 | 6.90 | 1.95 |
| Site 741 | C-I-6 | Geological | 19.70 | 39.34 | 8.85 | 5.28 | 1.74 |  | 1.63 | 1.65 | 3.46 |
| Site 741 | C-I-8 | Geological | 4.89 | 6.50 | 6.90 | 6.10 | 2.20 |  |  | 1.91 | 4.09 |
| Site 741 | C-I-9 | Geological | 4.38 | 5.76 | 12.99 | 7.39 | 3.02 | 5.29 | 2.03 | 2.72 | 4.77 |
| Site 741 | C-II-1 | Geological | 35.57 | 33.34 | 25.70 | 22.25 | 11.17 | 8.56 | 10.57 | 8.65 | 3.25 |
| Site 741 | C-II-2 | Geological | 27.22 | 32.97 | 32.15 | 26.01 | 13.81 | 14.60 | 13.19 | 9.60 | 5.93 |
| Site 741 | C-II-3 | Geological | 29.42 | 28.27 | 17.60 | 18.74 | 9.71 | 8.86 | 8.94 | 8.56 | 3.91 |
| Site 741 | C-II-4 | Geological | 38.50 | 46.13 | 21.99 | 20.59 | 9.79 | 14.57 | 9.76 | 8.29 | 11.70 |
| Site 741 | C-II-5 | Geological | 28.85 | 29.45 | 0.00 | 17.89 | 9.15 | 10.47 | 7.72 | 7.93 | 3.38 |
| Site 810 | D-I-2 | Geological | 30.86 | 17.70 | 7.10 | 3.58 | 1.43 | 1.14 |  | 1.13 | 2.17 |
| Site 810 | D-I-3 | Geological | 22.32 | 11.81 | 4.34 | 2.63 | 0.97 |  |  | 1.19 | 0.68 |
| Site 810 | D-I-4 | Geological | 24.44 | 14.66 |  | 3.11 | 1.57 |  | 2.44 | 2.91 | 1.14 |
| Site 810 | D-I-5 | Geological | 40.37 | 22.61 | 11.45 | 4.56 | 1.86 |  | 2.44 | 2.14 | 0.91 |
| Big Spring | E-I-1 | Geological | 1730.26 | 469.14 | 803.28 | 436.44 | 226.90 | 202.38 | 136.99 | 103.95 | 74.87 |
| Big Spring | E-I-2 | Geological | 2460.65 | 546.79 | 1302.58 | 619.56 | 325.23 | 275.68 | 182.52 | 133.40 | 96.50 |
| Big Spring | E-I-3 | Geological | 2024.81 | 513.02 | 1044.74 | 504.57 | 263.30 | 236.98 | 156.50 | 117.96 | 85.80 |
| Big Spring | E-I-4 | Geological | 861.48 | 403.38 | 416.56 | 217.45 | 112.29 | 101.88 | 71.14 | 56.34 | 41.10 |
| Big Spring | E-I-5 | Geological | 1374.12 | 463.12 | 636.69 | 337.04 | 174.38 | 153.68 | 103.25 | 79.06 | 57.22 |
| Big Spring | E-II-1 | Geological | 96.64 | 128.57 | 37.67 | 24.07 | 11.44 | 14.07 | 12.60 | 11.77 | 16.77 |
| Big Spring | E-II-2 | Geological | 97.99 | 139.12 | 36.95 | 24.43 | 11.00 | 19.34 | 10.98 | 12.16 | 13.89 |
| Big Spring | E-II-3 | Geological | 100.62 | 133.17 | 41.10 | 24.18 | 11.62 | 12.60 | 10.57 | 12.23 | 15.48 |
| Big Spring | E-II-4 | Geological | 101.03 | 132.90 | 38.89 | 23.97 | 11.48 | 10.25 | 11.38 | 12.70 | 13.89 |
| Big Spring | E-II-5 | Geological | 100.35 | 148.45 | 40.28 | 24.84 | 11.58 | 12.83 | 11.79 | 11.61 | 15.73 |
| Big Spring | E-III-1 | Geological | 107.69 | 62.94 | 32.49 | 19.04 | 9.43 | 13.10 | 10.98 | 10.64 | 10.04 |
| Big Spring | E-III-2 | Geological | 112.47 | 65.78 | 30.30 | 19.91 | 8.88 | 10.94 | 10.16 | 12.81 | 13.41 |
| Big Spring | E-III-3 | Geological | 108.14 | 63.33 | 29.20 | 19.01 | 8.83 | 11.83 | 10.16 | 11.29 | 14.71 |
| Big Spring | E-III-4 | Geological | 104.86 | 62.79 | 32.52 | 19.14 | 8.69 | 11.47 | 10.16 | 12.17 | 13.25 |
| Big Spring | E-III-5 | Geological | 104.25 | 60.62 | 32.91 | 18.23 | 8.86 | 10.97 | 10.16 | 12.70 | 13.60 |
| Big Spring | E-IV-1 | Geological | 112.62 | 71.56 | 38.76 | 19.00 | 8.56 | 7.56 | 10.16 | 10.79 | 13.67 |
| Big Spring | E-IV-2 | Geological | 117.59 | 77.19 | 33.24 | 19.66 | 9.08 | 10.03 | 10.16 | 10.99 | 11.10 |
| Big Spring | E-IV-3 | Geological | 115.20 | 77.52 | 38.53 | 18.93 | 8.61 | 9.00 | 9.35 | 9.53 | 12.07 |
| Big Spring | E-IV-4 | Geological | 116.10 | 75.40 | 35.15 | 18.06 | 8.54 | 10.61 | 10.16 | 10.79 | 10.30 |
| Big Spring | E-IV-5 | Geological | 113.09 | 76.93 | 37.31 | 18.76 | 8.46 | 11.30 | 8.54 | 11.70 | 11.34 |
| Truman Road site |  | Archaeological | 178.33 | 85.59 | 68.89 | 50.34 | 26.70 | 25.45 | 22.06 | 13.02 | 10.69 |
| Truman Road site |  | Archaeological | 15.71 | 14.70 | 24.72 | 98.80 | 96.94 | 146.47 | 111.58 | 65.71 | 56.22 |
| Truman Road site |  | Archaeological | 72.49 | 63.77 | 73.93 | 87.62 | 55.77 | 66.42 | 44.62 | 20.55 | 13.98 |
| Truman Road site |  | Archaeological | 20.77 | 12.34 | 14.00 | 9.32 | 5.67 | 7.35 | 5.31 | 3.81 | 4.47 |
| Truman Road site |  | Archaeological | 10.19 | 14.72 | 16.60 | 28.24 | 20.67 | 43.52 | 34.25 | 19.04 | 16.42 |
| Truman Road site |  | Archaeological | 13.19 | 12.02 | 17.36 | 15.89 | 9.31 |  |  | 4.01 | 7.80 |
| Truman Road site |  | Archaeological | 89.45 | 103.67 | 126.13 | 140.50 | 88.35 | 141.49 | 114.31 | 115.52 | 103.70 |
| Truman Road site |  | Archaeological | 102.90 | 96.19 | 102.03 | 96.70 | 30.62 | 40.59 | 34.37 | 30.44 | 19.76 |
| Truman Road site |  | Archaeological | 49.69 | 42.10 | 32.31 | 45.65 | 26.66 | 26.86 | 19.59 | 12.01 | 14.55 |
| Truman Road site |  | Archaeological | 50.05 | 30.07 | 38.14 | 39.06 | 21.74 | 32.91 | 25.94 | 20.64 | 14.35 |
| Truman Road site |  | Archaeological | 176.66 | 173.75 | 178.41 | 174.30 | 118.21 | 196.06 | 164.52 | 122.19 | 106.26 |
| Truman Road site |  | Archaeological | 152.73 | 142.23 | 104.12 | 161.51 | 143.87 | 418.88 | 410.86 | 361.70 | 258.58 |
| Truman Road site |  | Archaeological | 3475.13 | 2022.69 | 1318.38 | 836.86 | 448.85 | 341.04 | 214.66 | 99.75 | 61.06 |
| Truman Road site |  | Archaeological | 83.90 | 66.36 | 74.11 | 110.55 | 65.13 | 45.89 | 25.44 | 9.92 | 14.55 |
| Truman Road site |  | Archaeological | 52.81 | 49.37 | 41.37 | 35.79 | 19.57 | 13.00 | 8.17 | 9.40 | 9.07 |
| Truman Road site |  | Archaeological | 56.15 | 47.41 | 26.11 | 27.43 | 11.24 | 10.94 | 10.51 | 8.57 | 17.07 |
| Truman Road site |  | Archaeological | 315.81 | 137.17 | 157.75 | 131.84 | 68.86 | 61.44 | 43.09 | 18.62 | 28.17 |
| Truman Road site |  | Archaeological | 229.86 | 761.54 | 376.74 | 359.10 | 192.13 | 104.31 | 59.66 | 18.55 | 26.67 |
| Truman Road site |  | Archaeological | 97.27 | 41.73 | 46.70 | 45.06 | 27.19 | 38.55 | 30.47 | 24.39 | 17.68 |
| Truman Road site |  | Archaeological | 43.86 | 61.26 | 70.27 | 108.56 | 52.18 | 58.19 |  | 13.21 | 47.36 |
| Truman Road site |  | Archaeological | 107.81 | 112.83 | 139.87 | 133.78 | 79.31 | 81.31 | 39.85 | 28.60 | 19.39 |
| Truman Road site |  | Archaeological | 44.27 | 38.07 | 26.88 | 38.47 | 13.80 | 14.80 | 6.73 | 7.55 | 5.04 |
| Truman Road site |  | Archaeological | 53.16 | 103.23 | 145.16 | 199.51 | 129.54 | 133.14 | 45.50 | 41.29 | 40.85 |
| Truman Road site |  | Archaeological | 131.19 | 120.56 | 133.43 | 150.98 | 93.96 | 103.80 | 58.53 | 40.61 | 43.29 |
| Truman Road site |  | Archaeological | 157.62 | 54.06 | 86.45 | 72.57 | 42.10 | 47.27 | 34.41 | 22.11 | 25.12 |
| Truman Road site |  | Archaeological | 29.02 | 21.95 | 12.39 | 9.02 | 4.90 | 8.50 |  | 8.71 | 10.16 |
| Solto Site (1) |  | Archaeological | 2.56 | 3.16 |  | 3.37 |  |  |  |  | 1.26 |
| Hayden Site |  | Archaeological | 83.97 | 79.15 | 81.75 | 76.24 | 39.43 | 46.76 | 31.28 | 14.60 | 32.85 |
| Hayden Site |  | Archaeological | 248.49 | 346.99 | 101.22 | 64.10 | 36.96 | 29.54 |  | 20.79 | 15.12 |
| Gateway Site |  | Archaeological | 24.87 | 20.54 | 12.98 | 14.97 | 9.41 | 8.78 | 7.38 | 7.21 | 6.46 |
| Gateway Site |  | Archaeological | 35.33 | 61.50 | 31.96 | 34.11 | 9.01 | 16.65 | 4.76 | 8.45 |  |
| Truman Road site |  | Archaeological | 1.97 |  |  |  |  |  |  |  |  |
| Truman Road site |  | Archaeological | 101.75 | 77.20 | 48.64 | 41.10 | 21.33 | 26.08 | 27.13 | 19.70 | 25.61 |
| Truman Road site |  | Archaeological | 47.03 | 40.93 | 24.00 | 26.24 | 8.15 | 13.16 | 5.52 | 4.19 | 3.66 |
| Truman Road site |  | Archaeological | 52.00 | 66.67 | 85.61 | 67.80 | 42.45 | 41.72 | 27.31 | 11.08 | 15.89 |
| Truman Road site |  | Archaeological | 532.55 | 409.07 | 280.92 | 187.97 | 112.27 | 110.19 |  | 49.83 | 37.89 |
| Lawless Site |  | Archaeological | 26.92 | 23.62 | 21.18 | 15.99 | 6.94 | 8.32 | 6.52 | 4.84 | 8.13 |
| Lawless Site |  | Archaeological | 151.93 | 106.02 | 61.93 | 41.06 | 19.45 | 13.68 | 10.95 | 6.93 | 5.85 |
| Snyders site | 11C8 | Archaeological | 76.96 | 61.85 | 54.87 | 49.07 | 19.67 | 14.59 | 14.70 | 6.83 |  |
| Snyders site | 11C8 | Archaeological | 86.64 | 39.48 | 55.98 | 51.21 | 25.53 | 21.79 | 29.44 | 20.70 | 18.81 |
| Snyders site | 11C8 | Archaeological | 2.16 | 2.29 |  |  |  |  |  |  |  |
| Snyders site | 11C8 | Archaeological | 57.31 | 35.69 | 25.05 | 18.37 | 8.54 |  | 6.91 | 3.80 | 8.16 |
| Snyders site | 11C8 | Archaeological | 46.67 | 39.94 |  | 26.11 | 13.04 | 14.58 | 16.86 | 12.82 | 19.52 |
| Snyders site | 11C8 | Archaeological | 46.19 | 36.32 | 30.89 |  | 6.50 | 7.32 | 9.55 | 5.12 |  |
| Loy site | 11GE692 | Archaeological | 63.38 | 54.97 | 39.37 | 33.57 | 10.11 | 10.30 | 10.10 | 7.14 | 7.39 |
| Loy site | 11GE692 | Archaeological | 34.69 | 19.77 | 24.39 | 24.63 | 8.49 | 8.25 | 6.50 | 3.43 | 0.00 |
| Loy site | 11GE692 | Archaeological | 455.78 | 416.33 | 366.54 | 159.42 | 70.32 | 30.20 | 34.10 | 21.65 | 21.50 |
| Loy site | 11GE692 | Archaeological | 100.46 | 61.18 | 55.08 | 42.45 | 25.94 | 33.86 | 38.89 | 24.91 | 27.54 |
| Loy site | 11GE692 | Archaeological | 83.16 | 39.33 | 34.67 | 24.94 | 9.85 | 26.94 | 9.78 | 6.37 | 11.53 |
| Loy site | 11GE692 | Archaeological | 28.25 | 32.32 | 15.96 | 20.79 | 10.17 | 10.14 | 13.70 | 10.65 |  |
| Wheeler site |  | Archaeological | 103.76 | 53.11 | 38.15 | 33.16 | 16.17 | 20.62 | 23.01 | 14.28 | 11.92 |
| Wheeler site |  | Archaeological | 962.53 | 582.01 | 477.81 | 299.94 | 174.53 | 117.54 | 103.94 | 49.52 | 47.97 |
| Wheeler site |  | Archaeological | 45.49 | 34.69 | 27.28 | 26.85 | 15.44 | 19.47 | 18.70 | 9.29 | 15.64 |
| Wheeler site |  | Archaeological | 147.73 | 60.06 | 95.32 | 82.36 | 51.20 | 54.03 | 50.82 | 42.69 | 43.76 |
| Wheeler site |  | Archaeological | 96.73 | 75.63 | 62.19 | 71.45 | 46.12 | 39.81 | 31.33 | 12.57 | 10.91 |
| Wheeler site |  | Archaeological | 68.22 | 58.73 |  | 27.56 | 8.32 | 5.47 | 9.68 | 7.35 | 23.58 |
| Priest Site |  | Archaeological | 62.37 | 55.52 | 72.27 | 86.07 | 47.91 | 23.55 | 21.56 | 6.54 | 9.40 |
| Priest Site |  | Archaeological | 63.02 | 68.64 | 45.95 | 51.90 | 33.13 | 28.19 | 27.27 | 9.81 | 8.36 |
| Priest Site |  | Archaeological | 6.09 |  |  | 5.17 | 3.27 |  | 1.76 | 0.87 |  |
| Priest Site |  | Archaeological | 294.28 | 113.29 | 176.90 | 131.86 | 78.79 | 68.46 | 59.31 | 29.00 | 27.25 |
| Priest Site |  | Archaeological | 10.18 | 9.88 | 8.86 |  | 3.05 |  | 3.20 | 1.68 |  |
| Priest Site |  | Archaeological | 181.70 | 133.09 | 86.08 | 46.13 | 27.00 | 22.71 | 19.83 | 13.52 | 14.38 |
| Priest Site |  | Archaeological | 56.76 | 49.20 | 19.58 | 18.94 | 9.94 | 10.26 | 8.39 | 5.05 | 4.45 |
| Solto Site | 23SC12 | Archaeological | 31.21 | 12.46 | 10.50 | 12.30 | 4.24 |  | 2.84 | 2.80 | 7.46 |
| Solto Site | 23SC12 | Archaeological | 46.60 | 42.00 | 28.13 | 30.53 | 9.08 | 5.71 | 8.04 | 5.01 |  |
| Solto Site | 23SC12 | Archaeological | 49.30 | 57.71 | 81.13 | 102.45 | 63.68 | 60.51 | 62.91 | 40.40 | 40.03 |
| Solto Site | 23SC12 | Archaeological | 62.83 | 46.64 | 41.00 |  | 14.12 | 13.12 | 15.97 | 10.08 |  |
| Solto Site | 23SC12 | Archaeological | 8.72 | 6.75 | 5.74 |  |  |  | 1.93 | 1.19 |  |
| Solto Site | 23SC12 | Archaeological | 25.86 | 18.81 | 15.50 | 20.80 | 12.18 | 13.48 | 13.48 | 9.70 | 9.15 |
| Verkamp Shelter | 23PH21 | Archaeological | 9.53 | 9.06 |  |  |  |  | 0.48 |  |  |
| Verkamp Shelter | 23PH21 | Archaeological | 68.27 | 41.77 | 23.67 | 17.10 | 7.11 |  | 6.86 | 5.79 | 5.26 |
| Verkamp Shelter | 23PH21 | Archaeological | 22.15 | 10.19 | 14.56 | 11.92 | 3.55 |  | 2.72 | 1.87 | 0.00 |
| Verkamp Shelter | 23PH21 | Archaeological | 3.81 | 5.83 |  | 3.64 | 1.87 |  | 1.48 | 1.15 |  |
| Verkamp Shelter | 23PH21 | Archaeological | 3.79 | 2.89 | 1.37 | 1.48 | 0.65 | 0.32 | 0.51 | 0.50 | 0.72 |
| Verkamp Shelter | 23PH21 | Archaeological | 37.46 | 48.51 |  | 13.13 | 5.65 |  | 2.65 | 1.02 |  |
| Verkamp Shelter | 23PH21 | Archaeological | 35.92 | 45.40 | 34.63 | 32.20 | 19.93 | 18.29 | 16.48 | 10.68 | 10.79 |
| Verkamp Shelter | 23PH21 | Archaeological | 5.14 | 3.16 |  | 1.39 | 0.89 | 0.85 | 1.42 | 0.61 | 0.94 |
| Verkamp Shelter | 23PH21 | Archaeological | 32.76 | 28.92 | 13.57 | 9.29 | 3.43 |  | 1.72 | 1.70 | 4.05 |
| Verkamp Shelter | 23PH21 | Archaeological | 15.78 | 7.31 | 5.70 | 4.39 | 1.67 |  | 2.70 | 2.87 | 3.77 |
| Verkamp Shelter | 23PH21 | Archaeological | 34.31 | 24.68 | 14.52 | 13.27 | 6.91 | 0.00 | 5.30 | 4.53 | 6.90 |
| Verkamp Shelter | 23PH21 | Archaeological | 39.39 | 20.05 | 20.25 | 12.67 | 6.54 | 10.81 | 11.19 | 6.37 | 8.70 |
| Verkamp Shelter | 23PH21 | Archaeological | 4.80 | 2.86 |  | 2.67 |  |  |  |  |  |
| Verkamp Shelter | 23PH21 | Archaeological | 22.37 | 20.10 | 21.09 | 20.94 | 14.36 | 10.57 | 9.81 | 4.05 | 3.55 |
| Verkamp Shelter | 23PH21 | Archaeological | 15.85 | 11.81 | 9.68 | 8.11 | 2.18 |  | 2.34 | 1.54 |  |
| Verkamp Shelter | 23PH21 | Archaeological | 21.51 | 12.58 | 5.38 | 3.19 | 2.02 | 0.73 |  | 0.85 | 0.56 |
| Verkamp Shelter | 23PH21 | Archaeological | 10.20 | 8.12 |  | 4.76 | 1.23 |  | 2.00 | 1.38 |  |
| Verkamp Shelter | 23PH21 | Archaeological | 32.11 | 21.93 | 7.20 | 9.57 | 3.62 |  | 2.86 | 2.04 |  |
| Verkamp Shelter | 23PH21 | Archaeological | 8.78 | 5.08 | 4.92 | 3.13 | 1.04 |  | 0.50 |  |  |
| Verkamp Shelter | 23PH21 | Archaeological | 29.22 | 33.14 | 28.28 | 15.61 | 7.29 | 3.64 | 1.82 | 0.94 |  |
| Verkamp Shelter | 23PH21 | Archaeological | 2.47 | 3.22 |  | 1.68 |  |  | 0.52 | 0.55 |  |
| Verkamp Shelter | 23PH21 | Archaeological | 23.28 | 21.32 | 14.82 | 7.33 | 1.98 |  | 2.24 | 0.30 |  |
| Verkamp Shelter | 23PH21 | Archaeological | 60.00 | 53.86 | 28.07 | 19.28 | 9.95 | 5.17 | 7.22 | 3.35 | 3.01 |
| Verkamp Shelter | 23PH21 | Archaeological | 71.48 | 51.19 | 32.55 | 21.23 | 9.82 | 1.45 | 4.55 | 1.28 |  |
| Verkamp Shelter | 23PH21 | Archaeological | 16.00 | 12.33 | 12.02 | 8.87 | 2.15 |  | 1.66 | 1.78 |  |
| Verkamp Shelter | 23PH21 | Archaeological | 21.86 | 17.75 | 13.71 | 9.21 | 5.20 | 6.07 | 3.72 | 2.86 | 4.25 |
| Verkamp Shelter | 23PH21 | Archaeological | 7.12 | 5.14 | 9.42 | 5.41 | 1.84 |  | 1.27 | 1.56 |  |
| Verkamp Shelter | 23PH21 | Archaeological | 16.23 | 19.94 | 14.42 | 13.38 | 6.95 | 5.63 | 7.07 | 6.81 | 7.46 |
| Verkamp Shelter | 23PH21 | Archaeological | 26.66 | 17.43 | 14.82 | 11.35 | 5.93 | 3.76 | 4.68 | 3.15 | 5.33 |
| Verkamp Shelter | 23PH21 | Archaeological | 51.81 | 43.57 | 29.76 | 20.58 | 10.63 | 9.63 | 8.39 | 4.42 | 6.12 |
| Verkamp Shelter | 23PH21 | Archaeological | 2.73 | 1.69 |  | 2.25 |  |  | 0.50 |  |  |
